# Silencing of LINC00461 enhances radiosensitivity of lung adenocarcinoma cells by down‐regulating HOXA10 via microRNA‐195

**DOI:** 10.1111/jcmm.14859

**Published:** 2020-01-22

**Authors:** Jiqiu Hou, Yanjun Wang, Hongmei Zhang, Yuxin Hu, Xiuqin Xin, Xiaodan Li

**Affiliations:** ^1^ Department of Pharmacy The Second Hospital of Jilin University Changchun China; ^2^ Department of Nursing The Second Hospital of Jilin University Changchun China; ^3^ Department of Pharmacy The First Hospital of Jilin University Changchun China; ^4^ Department of Pulmonary and Critical Care Medicine The Second Hospital of Jilin University Changchun China; ^5^ Department of Pulmonary and Critical Care Medicine The First Hospital of Jilin University Changchun China

**Keywords:** homeoboxa10, long non‐coding RNA LINC00461, lung adenocarcinoma, microRNA‐195, radiosensitivity

## Abstract

Lung adenocarcinoma is recognized as one of the most recurrent tumours in adults. Long non‐coding RNAs (lncRNAs) are non–protein‐coding transcripts and have been demonstrated to regulate biological functions during tumorigenesis. Our study aims to investigate the underlying molecular mechanisms of LINC00461/microRNA‐195 (miR‐195)/HOXA10 responsible for its involvement in lung adenocarcinoma. We firstly selected differentially expressed lncRNAs and genes by the Cancer Genome Atlas (TCGA) database and Gene Expression Omnibus (GEO). The functional role of LINC00461 in lung adenocarcinoma was then determined using ectopic expression, knockdown and reporter assay experiments. Besides, we detected the expression profiles of LINC00461, miR‐195, HOXA10 and apoptosis‐ and invasion‐related genes. Cell proliferation, migration and invasion were evaluated. In vivo tumour formation ability was analysed. Overexpressed LINC00461 and HOXA10 but down‐regulated miR‐195 were observed in primary and metastatic lung adenocarcinoma. LINC00461 negatively regulated miR‐195, while miR‐195 negatively regulated HOXA10. Forced LINC00461 expression decreased expression of miR‐195 and Bax, increased expression of HOXA10, MMP‐2, MMP‐9 and Bcl‐2, promoted cell proliferation, migration and invasion as well as tumour formation, and enhanced radiosensitivity of lung adenocarcinoma cells. However, these effects were reversed by lentivirus‐mediated miR‐195–forced expression, thereby suggesting that miR‐195 could antagonize the harmful effect of LINC00461 on lung adenocarcinoma cells. Collectively, the present study provides evidence supporting the inhibitory effect of LINC00461 silencing on lung adenocarcinoma, which suppresses lung adenocarcinoma cell migration, invasion and radiosensitivity via HOXA10 by binding to miR‐195, which provides a promising basis for the targeted intervention treatment for human lung adenocarcinoma.

## 1 INTRODUCTION

Lung cancer is among the most prevalent malignancies worldwide associated with a clinically high overall cancer‐related mortality.^1^ Lung adenocarcinoma, presenting as epithelial cancer of glandular origin, has the highest prevalence of all types of non‐small cell lung cancer (NSCLC).^2^ In addition, lung adenocarcinoma accounts for 40% of lung cancer cases, accounting for over 500,000 yearly deaths worldwide.^3^ Approximately 25%‐30% of patients with NSCLC have contracted a localized disease because of surgical intervention for curative purposes; unfortunately, the 5‐year survival rate still remains to be poor, and 40%‐70% of these patients are at risk of a susceptible systemic disease with or without local relapses and could eventually die from it.^4^ In order to improve the prognosis of patients with lung adenocarcinoma, the identification of molecular mechanism is required.

Among the non‐coding RNAs, long non‐coding RNAs (lncRNAs) are transcripts with more than 200 nucleotides in length.^5^ Aberrant lncRNA expression is involved in various types of human cancers, including breast cancer, prostate cancer and colorectal cancer.^6^ LINC00461, a human homolog of the mouse lncRNA C130071C03Rik, is reported to present with an overexpressed expression profile in human glioma tissues.^7^ Furthermore, LINC00461 was predicted to be up‐regulated in lung adenocarcinoma in the Cancer Genome Atlas (TCGA). LncRNAs binding with microRNAs (miRNAs or miRs) inhibit expression of miRs by binding to their target sites, thereby regulating the protein expression.^8^ Moreover, a biological prediction website RNA22 predicted LINC00461 to directly regulate miR‐195. MiR‐195, located on the chromosome 17p13.1, is a member of the miR‐16/15/195/424/497 family.^9^ Besides, miR‐195 has been reported to be involved in tumorigenesis as a tumour suppressor.^10^ High miR‐195 level has been used as a tumour predictor for the prognosis of non‐smoking women with lung adenocarcinoma.^11^ A bioinformatics website microRNA.org then further revealed that homeobox A10 (HOXA10) was a potential target gene of miR‐195. HOXA10 belongs to the homeobox gene family that is well conserved during evolution and plays a vital role in several biological processes.^12^ A competing endogenous RNA network analysis showed that lncRNA ENSG00000240990 competed with HOXA10 to affect the prognosis of lung adenocarcinoma patients.^13^ Furthermore, HOXA10 with recurrent up‐regulation has been documented in human lung cancer cells and tissues.^14^ Base on the aforementioned literature, we hypothesized that LINC00461 may be involved in lung adenocarcinoma by regulating HOXA10 via miR‐195. Therefore, this study was planned to explore the regulatory mechanism of the LINC00461/miR‐195/HOXA10 regulatory network in lung adenocarcinoma.

## 2 MATERIALS AND METHODS

### 2.1 Microarray‐based gene expression profiling

TCGA (http://cancergenome.nih.gov/) was used to retrieve gene expression datasets related to lung adenocarcinoma, and the transcriptome profiling data containing the package edgeR of R was analysed using differential analysis.^15^ False‐positive discovery (FDR) correction was performed on the p‐value with package multitest. FDR < 0.05 and |log2 (fold change)| > 2 were set as the threshold to screen DEGs (differentially expressed genes). The Gene Expression Omnibus (GEO) (http://www.ncbi.nlm.nih.gov/geo) was employed to retrieve gene expression dataset (http://www.ncbi.nlm.nih.gov/geo/query/acc.cgi?acc=GSE27716) relevant to glioma and annotation probe files. The chips were obtained upon detection of GPL570 ([HG‐U133_Plus_2] Affymetrix Human Genome U133 Plus 2.0 Array). The Affy package of R software was employed for background correction and normalization of each chip data.^16^ The linear empirical Bayes statistical method in the Limma installation package, in combination with the traditional *t* test, was employed for non‐specific filtration of the expression data, in order to screen the differentially expressed RNAs and genes.^17^ The miRNAs interacting with specific lncRNA and gene were determined using RNA22 (https://cm.jefferson.edu/rna22/).

### 2.2 Cell culture

Lung adenocarcinoma cell lines H1299, A549, PC9, LTEP‐A‐2, NCI‐H1650 and MRC‐5 were acquired from the Institute of Biochemistry and Cell Biology, Shanghai Institutes for Biological Sciences, Chinese Academy of Sciences (Shanghai, China). After cell recovery, the cells were cultured in Dulbecco's Modified Eagle's Medium (DMEM) containing 10% foetal bovine serum (FBS) in a humidified incubator at 37°C with 5% CO_2_. Upon reaching 90% confluence, the cells were treated with 0.25% trypsin (T1300, Beijing Solarbio Science & Technology Co Ltd) for subculture (1:3). Cells presenting with a high expression of LINC00461 and HOXA10 and a low expression of miR‐195 were selected for subsequent experiments.

### 2.3 Dual‐luciferase reporter gene assay

A biological prediction website RNA22 was employed to predict the target relationship between LINC00461 and miR‐195. The 3’untranslated region (3’UTR) of LINC00461 was amplified, and PCR products were subcloned and ligated into the pmirGLO (Promega) using the endonuclease sites SpeI and Hind III to collectively construct pMIR‐LINC00461‐wild‐type (Wt). Then, the LINC00461 binding site mutant (Mut) (LINC00461‐Mut: GACCAGGGACGCTGCTC.) was predicted using the target gene database, and the recombinant vector was constructed by the T4 DNA Ligase. MiR‐195 mimic and negative control (NC) were, respectively, cotransfected using the luciferase reporter vector into NCI‐H1650 cells (with Renilla luciferase vector pRL‐TK [Takara Biotechnology Ltd] as internal control). After 48 hours, the cells were collected and lysed, and the relative luciferase activity was measured using the Dual‐Luciferase Reporter Assay System (Promega). The experiment was performed three times independently.

A biological prediction website microRNA.org was employed to predict the target relationship between miR‐195 and HOXA10. The 3’UTR of HOXA10 was amplified, and the PCR products were subcloned and ligated into pmirGLO (Promega) using the endonuclease sites SpeI and Hind III to conjointly construct pMIR‐HOXA10‐Wt. Then, the HOXA10 binding site Mut (auUAAUAUUGUAAACGACCUg) was predicted by the target gene database and the recombinant vector was constructed using T4 DNA Ligase. MiR‐195 mimic and NC were, respectively, cotransfected with the luciferase reporter vector into NCI‐H1650 cells (with Renilla luciferase vector pRL‐TK [Takara Biotechnology Ltd] as internal control). After 48 hours, the cells were collected and lysed, and the relative luciferase activity was measured using the Dual‐Luciferase Reporter Assay System (Promega). The experiment was performed three times independently.

### 2.4 RNA fluorescence in situ hybridization (FISH)

FISH technique was employed to identify the subcellular localization of LINC00461 in the cells. According to the instructions of Ribo™ lncRNA FISH Probe Mix (Red) (Ribo Biological), coverslips were placed in 6‐well plates, and cells in logarithmic growth phase were seeded in the plates for 1 d to facilitate cell confluence to 80%. Then, the coverslips were removed, rinsed with phosphate‐buffered saline (PBS), fixed using 1 mL of 4% paraformaldehyde, followed by the addition of protease K (2 μg/mL), glycine and acetylation reagent, and then finally incubated in 250 μL pre‐hybridization solution for 1 hour at 42ºC. Next, the pre‐hybridization solution was removed, and 250 μL of pre‐hybridization solution containing probes (300 ng/mL) was added the samples for overnight incubation at 42°C. Then, the coverslips were rinsed 3 times with phosphate‐buffered saline with Tween‐20 (PBST), stained using 4',6‐diamidino‐2‐phenylindole (DAPI) diluted with PBST (1:800) in 24‐well plates for 5 minutes, then rinsed with PBST 3 times (each time 3 minutes) repeatedly and sealed with fluorescence quenching agent. Five different visual fields were randomly selected for observation and photograph under a fluorescence microscope (Olympus Optical Co., Ltd).

### 2.5 RNA pull‐down assay

Using the Magnetic RNA‐Protein Pull‐Down Kit (Pierce), 1 μg of biotin‐labelled RNA LINC00461 was added into Eppendorf (EP) tubes, added with 500 μL of Structure Buffer at 95ºC for 2 minutes and incubated on ice for 3 minutes. Following suspension, 50 μL of magnetic beads was added to the EP tube for overnight incubation at 4ºC, followed by 1610 g centrifugation for 3 min. Afterwards, the precipitate was rinsed with 500 μL RIP 3 times, and then, 10 μL of cell lysate was added, and placed at room temperature for 1 hour. Then, the magnetic beads RNA‐protein complex was centrifuged to collect the supernatant followed by rinsing with 500 μL of RIP Wash Buffer 3 times. The 10 μL of supernatant was used as the Input of protein. The protein concentration was measured, and the protein expression was detected by conducting Western blot analysis. The experiment was performed three times independently.

### 2.6 RNA binding protein immunoprecipitation (RIP)

The RIP kit (Millipore) was used to identify the binding between LINC00461 and HOXA10. Cells were rinsed with pre‐cooled PBS, ice‐bathed in radioimmunoprecipitation assay (RIPA) lysis buffer (P0013B, Shanghai Beyotime Biotechnology Co. Ltd) for 5 minutes and centrifuged (35 068 *g*) at 4ºC for 5 minutes to collect the supernatant. Cell extract was used for the Input of protein and co‐precipitation with the antibody. A total of 50 μL of magnetic beads were suspended in 100 μL of RIP Wash Buffer, after which 5 μg antibody was added for incubation according to the grouping. After cleaning, the magnetic bead‐antibody complex was resuspended in 900 μL of RIP Wash Buffer, and incubated with 100 μL of cell extract at 4ºC overnight. The samples were placed on a magnetic base in order to collect the magnetic beads‐protein complex. Then, the samples and the Input were collected and digested using protease K for subsequent Western blot analysis. The antibody used in RIP was HOXA10 (ab32381, 1:2000, Abcam Inc) mixed for 30 minutes at room temperature, and IgG (1:100, ab172730, Abcam Inc) as NC. The experiment was performed three times independently.

### 2.7 Cell grouping and transfection

NCI‐H1650 cells in the logarithmic growth phase were selected for further experimentation. The collected cells were seeded in a 6‐well plate at a density of 5 × 10^4^ cells/well and cultured in fresh complete medium to attain a cell confluence of 50%‐80% for potential transfection. According to the instructions of Lipofectamine 2000 (11668‐027, Invitrogen), the cells were assigned into the following groups: Control (NCI‐H1650 cells without treatment), short hairpin RNA (shRNA)‐NC (NCI‐H1650 cells transfected with LINC00461 NC), LINC00461 + mimic NC (NCI‐H1650 cells transfected with LINC00461 overexpression and mimic NC), sh‐LINC00461 (NCI‐H1650 cells transfected with sh‐LINC00461), LINC00461 + miR‐195 mimic (NCI‐H1650 cells transfected with LINC00461 elevation and miR‐195 mimic); blank (NCI‐H1650 cells without treatment), NC (NCI‐H1650 cells transfected with unrelated sequence), miR‐195 mimic (NCI‐H1650 cells transfected with miR‐195 mimic), miR‐195 inhibitor (NCI‐H1650 cells transfected with miR‐195 inhibitor), sh‐HOXA10 (NCI‐H1650 cells transfected with sh‐HOXA10), and sh‐HOXA10 + miR‐195 mimic (NCI‐H1650 cells transfected with sh‐HOXA10 and miR‐195 mimic) groups. Lipo solution was prepared by 240 μL of serum‐free medium and 10 μL of lipo for incubation for 5 minutes. Plasmid solution was prepared by a combination of 20 μL of serum‐free medium and 4 μg of plasmid. The mixture prepared by combining the Lipo solution and the plasmid solution was placed at room temperature for 20 minutes, which was added into the well in a drop wise manner and mixed gently. Next, the cells were preserved in a 5% CO_2_ incubator at 37ºC for 5‐6 hours, and the complete medium replacement was conducted to sustain further culture for 24‐48 hours. The transfection efficiency was determined and the follow‐up experiments were conducted upon reaching over 70% transfection efficiency.

### 2.8 Isolation of mRNA and reverse transcription quantitative polymerase chain reaction (RT‐qPCR)

Trizol Reagent (Invitrogen) was used to extract total RNA, and the reverse transcription was conducted using the PrimeScript™ RT reagent Kit (RRO37A, TaKaRa Biotechnology Co. Ltd). Fluorescence quantitative PCR instrument (ABI 7500, Applied Biosystems) was employed for amplification of the target genes and reference genes. U6 was regarded as the internal reference for miR‐195, and glyceraldehyde‐3‐phosphate dehydrogenase (GAPDH) was regarded as the internal reference for others. On the basis of the 2^−ΔΔCT^ method, the expression of the genes was calculated.^18^ The experiment was performed three times independently. Primer sequences are shown in Table 1.

**Table 1:** The primer sequences for RT‐qPCR

Genes	Sequences
LINC00461	Forward: 5′‐GCGTGGACTACTCTGATG‐3′
	Reverse: 5′‐CCAAGTGCTTACTGTCT‐3′
miR‐195	Forward: 5′‐CGTAGCAGCACAGAAAT‐3′
	Reverse: 5′‐GTGCAGGGTCCGAGGT‐3′
HOXA10	Forward: 5′‐CTCGCCCATAGACCTGTGG‐3′
	Reverse: 5′‐GTTCTGCGCGAAAGAGCAC‐3′
MMP‐2	Forward: 5′‐TGACTTTCTTGGATCGGGTCG‐3′
	Reverse: 5′‐AAGCACCACATCAGATGACTG‐3′
MMP‐9	Forward: 5′‐AGACCTGGGCAGATTCCAAAC‐3′
	Reverse: 5′‐CGGCAAGTCTTCCGAGTAGT‐3′
Bax	Forward: 5′‐CATATAACCCCGTCAACGCAG‐3′
	Reverse: 5′‐GCAGCCGCCACAAACATAC‐3′
Bcl‐2	Forward: 5′‐TTGCCAGCCGGAACCTATG‐3′
	Reverse: 5′‐CGAAGGCGACCAGCAATGATA‐3′
GAPDH	Forward: 5'‐TGTGGGCATCAATGGATTTGG‐3'
	Reverse: 5'‐ACACCATGTATTCCGGGTCAAT‐3'
U6	Forward: 5'‐AAAGCAAATCATCGGACGACC‐3′
	Reverse: 5′‐GTACAACACATTGTTTCCTCGGA‐3′

Abbreviations: Bax, Bcl‐2‐associated X protein; Bcl‐2, B‐cell lymphoma 2; GADPH, glyceraldehyde‐3‐phosphate dehydrogenase; miR‐195, microRNA‐195; MMP, matrix metalloproteinase; RT‐qPCR, reverse transcription quantitative polymerase chain reaction.

### 2.9 Western blot analysis

Total protein was extracted using RIPA buffer containing phenylmethanesulfonyl fluoride (PMSF), incubated on ice for 30 minutes and centrifuged at 25 764 *g* at 4°C for 10 minutes to extract the supernatant. The concentration of respective proteins was determined using a bicinchoninic acid (BCA) Protein Assay Kit (23225, Pierce). A total of 50 μg loading sample was added in each well and separated using 10% sodium dodecyl sulphate polyacrylamide gel electrophoresis (P0012A, Beyotime Institute of Biotechnology) for 2 hours. Next, the proteins were transferred onto a polyvinylidene fluoride membrane (ISEQ00010, Millipore) for 2 hours. The membrane was then blocked with Tris‐buffered saline with Tween 20 (TBST) buffer containing 5% skimmed milk powder for 2 hours and incubated at 4ºC overnight with the following primary antibodies provided by Abcam: HOXA10 (1:1000, ab23392), matrix metalloproteinase (MMP‐2) (1:500, ab37150), MMP‐9 (1:1000, ab73734), B‐cell lymphoma 2‐associated X protein (Bax) (1:2000, ab32503) and B‐cell lymphoma 2 (Bcl‐2) (1:1000, ab32124), with β‐actin (1:1000, ab8227) as the internal reference. Then, the membrane was visualized using enhanced chemiluminescence (WBKLS0100, Millipore), followed by analysis using the Image J software (Bio‐Rad Laboratories). The experiment was performed three times independently.

### 2.10 Radiotherapy and 3‐(4, 5‐dimethylthiazol‐2‐yl)‐2, 5‐diphenyltetrazolium bromide (MTT) assay

The cells in the logarithmic growth phase were seeded in a 96‐well plate at a concentration of 1 × 10^5^ cells/mL. Cells were then allocated into the irradiation group and the control group, with 4 groups in each group, 8 wells in each group and 100 μL in each well, and cultured in cell incubator of 5% CO_2_ at 37°C. Cells were irradiated with 6 MV X‐ray using a linear accelerator, with X‐ray dose of 1, 1.5, 2 and 2.5 Gy to isocenter for each group, respectively. The dose rate was 100 Mu/min, and the irradiation field was 10 cm × 10 cm. The aforementioned steps were performed to optimize the dose of radiation.

After grouping, the cells underwent the same treatment except for the X‐ray dose of 2 Gy to isocenter. The control group received no treatment. Next, 2 mg/mL MTT liquid was added in each group for culture for 4 hours, and then, DMSO (Beijing Chemical Factory, China) liquid was added for oscillation for 10 minutes to fully dissolve the crystals. After cell culture for 24, 48 and 72 hours, the optical density (OD) value was measured using a microplate reader (680, Bio‐Rad Laboratories) at an excitation wavelength 490 nm. MTT curve was drawn with absorbance value as the ordinate and interval time as the abscissa. The survival cells (%) = A490 of the irradiation group/A490 of the control group × 100%. The experiment was performed three times independently.

### 2.11 Scratch test

Six‐well plates of six groups were selected and grouped in strict accordance with the corresponding cells. Behind each plate, a line was drawn every 0.5‐1 cm across the well using a marker pen. A minimum of five lines was drawn per well. Then, each well was added with approximately 5 × 10^5^ cells for overnight culture. On the second day, the pipette tip was used to vertically scratch the line. Cells were rinsed with PBS for three times with the removal of the scratched‐out cells and cultured with serum‐free medium in an incubator with 5% CO_2_ at 37ºC followed by observation at 0 and 24 hours under an inverted microscope (×40). Photographs were obtained, and scratch distance was measured. The experiment was performed three times independently.

### 2.12 Transwell assay

The apical chamber of each chamber was added with 60 μL of 50 mg/L Matrigel (dilution ratio of 1:8, Sigma‐Aldrich Chemical Company). After drying at room temperature, the remaining liquid in the plate was discarded. Next, each well was added with 50 μL serum‐free medium containing 10 g/L bovine serum albumin (BSA) and placed at 37ºC for 30 minutes. Cells in the logarithmic phase were adjusted to a density of 1 × 10^5^ cells/mL by the addition of serum‐free medium containing 10 g/L BSA, after which 200 μL of cell suspension was added to each chamber. Then, 500 μL of medium containing 100 mL/L FBS was added into the basolateral chamber of the 24‐well plate, which was then put in an incubator with 5% CO_2_ at 37ºC for 24 hours. Subsequently, the chamber was removed, and cells on the polyvinylidene fluoride (PVDF) membrane closing to the inner side of the chamber were wiped out using a cotton swab. Cells were fixed with 95% alcohol at room temperature for 30 minutes, stained with crystal violet (Sigma‐Aldrich Chemical Company) for 20 minutes and washed using water for three times. Photographs were obtained under an inverted microscope (CKX41SF, Sigma‐Aldrich Chemical Company) with the number counted. Each experiment was performed three times independently.

### 2.13 Cellular tumourigenicity in nude mice

A total of 24 specific pathogen‐free male nude mice (aged 5 weeks old and weighing 16‐18 g, SLAC Laboratory Animal Co., Ltd) were chosen for this experiment. Mice had free access to ordinary feed and drinking water in the condition with humidity of 45%‐50% and proper temperature of 25‐27ºC. Mice were grouped into four groups (control, shRNA‐NC, sh‐LINC00461, LINC00461) with each group comprising of 6 mice. Tumour cell inoculation was conducted after anaesthesia. Cells in the logarithmic growth phase were resuspended in 50% Matrigel (BD Biosciences) to adjust the cell concentration to 1 × 10^7^ cells/mL. Then, 0.5 mL of cell suspension was subcutaneously injected into the left armpit of mice (5 × 10^6^ cells). On the 1st, 2nd, 3rd, 4th and 5th week after tumour cell inoculation, the tumour length and width were measured using a digital caliper to calculate the tumour size with the formula of (L × W^2^)/2, in which the L is the length of the tumour, and the W is the width of the tumour. On the 4th week after tumour cell inoculation, the mice were killed by CO_2_ for tumour sample collection. Samples were initially fixed in 10% formaldehyde solution for 24 hours, dewaxed twice using xylene for 10 minutes each time and then treated with gradient ethanol of variable concentrations (100% ethanol for 5 minutes, 90% ethanol for 2 minutes and 70% ethanol for 2 minutes). Then, the samples were stained using haematoxylin for 7 minutes, treated with 95% ethanol for 5 seconds and counterstained using eosin for 1 minute, respectively. Next, the samples were hydrated twice with gradient ethanol of variable concentrations (100%, 95%, 75% and 50%) for 2 minutes each time, cleared twice using xylene for 5 minutes each time and dried. Finally, the samples were sealed and observed under an optical microscope. The experiment was performed three times independently.

### 2.14 Statistical analysis

SPSS 21.0 software (IBM Corp.) was applied to analyse data and calculate mean value and standard deviation. Data between two groups were compared by unpaired *t* test or paired *t* test, and corrected by Welch test. Comparisons of measurement data among multiple groups were analysed by one‐way analysis of variance (ANOVA), and comparisons of mean values of samples between two groups were analysed by least significant difference (LSD). Data at different time points were compared with repeated measurement ANOVA *P* < .05 was an indicative of significant difference.

## 3 RESULTS

### 3.1 LINC00461 is overexpressed in lung adenocarcinoma

**Figure 1:**
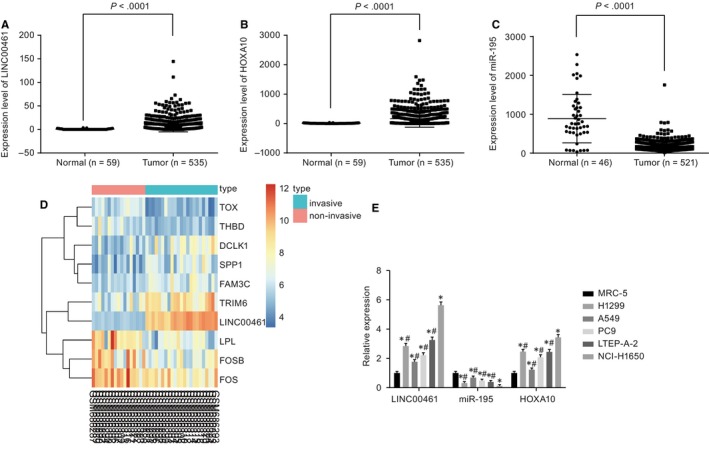
LINC00461 and HOXA10 are highly expressed while miR‐195 is poorly expressed in lung adenocarcinoma. A, LINC00461 is overexpressed in lung adenocarcinoma from the TCGA database. B, HOXA10 is overexpressed in lung adenocarcinoma from the TCGA database. C, miR‐195 is poorly expressed in lung adenocarcinoma from the TCGA database. D, A heat map of http://www.ncbi.nlm.nih.gov/geo/query/acc.cgi?acc=GSE27716; the top bar represents the types of samples, the blue represents invasive tumour samples, the red represents non‐invasive tumour samples, and colour gradation in upper right represents gene expression (from top to bottom, the colour changes from red to green, which says the expression level from high to low); the abscissa denotes the sample number, the vertical axis represents the gene name, each box in the diagram represents expression of a gene in a sample, and the left side of the tree graph represents a cluster analysis based on the differences of gene expression. E, mRNA levels of LINC00461, miR‐195 and HOXA10 in lung adenocarcinoma cell lines. The x‐axis and y‐axis in D represent the sample number and the differentially expressed genes, respectively. *P < .05, vs MRC‐5 cell line. ^#^ *P* < .05 vs NCI‐H1650 cell line. The experimental data of RT‐qPCR were expressed as mean value ± standard deviation, and the expression of multiple cell lines was analysed by one‐way ANOVA. The experiment was run in triplicate independently. miR‐195, microRNA‐195; HOXA10, Homeobox A10; ANOVA, analysis of variance; and RT‐qPCR, reverse transcription quantitative polymerase chain reaction

TCGA database demonstrated a high expression of LINC00461 and HOXA10 in lung adenocarcinoma (Figure 1A,B), while miR‐195 had poor expression in lung adenocarcinoma (Figure 1C). Microarray data of http://www.ncbi.nlm.nih.gov/geo/query/acc.cgi?acc=GSE27716 further confirmed the high expression of LINC00461 in metastatic lung adenocarcinoma (Figure 1D). Results of the LINC00461 expression, miR‐195 expression and HOXA10 mRNA expression in H1299, A549, PC9, LTEP‐A‐2 and NCI‐H1650 cell lines and MRC‐5 cell line (pulmonary fibroblast line) showed that, when compared to MRC‐5 cell line, the expression of LINC00461 and HOXA10 significantly increased while the miR‐195 expression decreased markedly in the other five cell lines (*P* < .05). In addition, LINC00461 and HOXA10 expressions were lower, while the miR‐195 expression was higher in H1299, A549, PC9 and LTEP‐A‐2 cell lines than those observed in the NCI‐H1650 cells (*P* < .05) (Figure 1E). Therefore, the NCI‐H1650 and PC9 cell lines were selected for subsequent experimentation.

### 3.2 LINC00461 up‐regulated HOXA10 by binding to miR‐195

**Figure 2:**
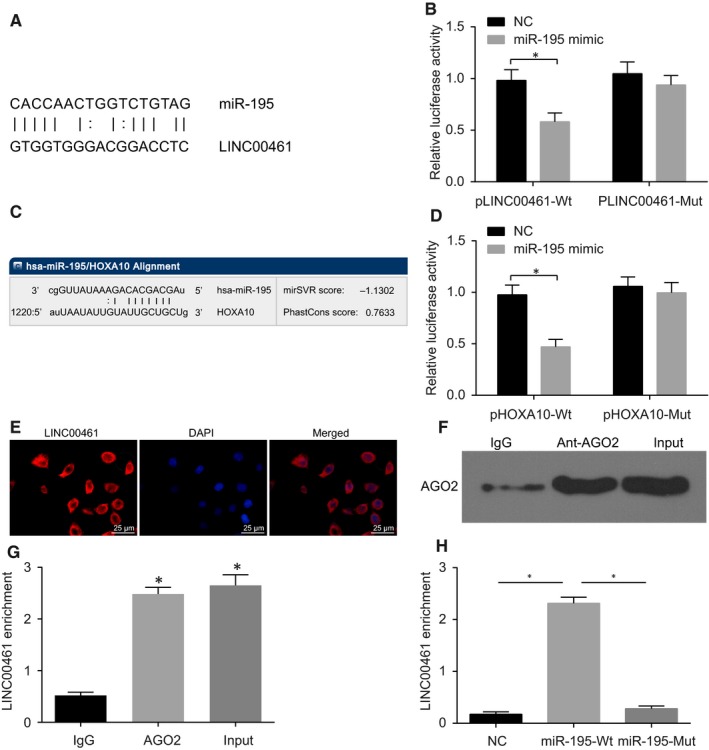
LINC00461 up‐regulates HOXA10 by binding to miR‐195. A, LINC00461 is presumed to bind to the 3'untranslated regions (3'UTR) of miR‐195 using RNA22. B, A combination of miR‐195 mimic and pLINC00461‐Wt significantly reduces the luciferase activity, suggesting that LINC00461 binds to the 3'UTR of miR‐195 (*P < .05, vs the control group). C, miR‐195 is presumed to bind to the 3'untranslated regions (3'UTR) of HOXA10 using RNA22; D, a combination of miR‐195 mimic and pHOXA10‐Wt significantly reduces the luciferase activity, suggesting that LINC00461 binds to the 3'UTR of HOXA10 (*P < .05, vs the control group); E, LINC00461 is predominantly localized in the cytoplasm observed by FISH test. F, Ago2 expression is detected by Western blot analysis (*P < .05, vs the IgG group). G, LINC00461 directly bound to Ago2 by RIP assay. H, LINC00461 binds to miR‐195, determined by RNA pull‐down test (*P < .05, vs the miR‐195‐Wt group). The results of luciferase activity, Western blot analysis and RNA pull‐down test were expressed as mean value ± standard deviation. Comparisons between two groups were conducted by *t* test. The experiment was run in triplicate independently. The comparisons among three groups were conducted by one‐way ANOVA. miR‐195, microRNA‐195; ANOVA, analysis of variance; Wt, wild‐type; HOXA10, Homeobox A10; and Mut, mutant type

Based on the bioinformatics website, we found that there was a specific binding point between miR‐195 and LINC00461 (Figure 2A). Dual‐luciferase reporter gene assay (Figure 2B) showed that when cotransfected with pLINC00461‐Wt, the luciferase activity of miR‐195 mimic group was lower than that of NC group (*P* < .05), while when cotransfected with pLINC00461‐Mut, no significant difference in the luciferase activity was observed (*P* > .05). The results suggested that LINC00461 competitively bound to miR‐195. Furthermore, HOXA10 was predicted as a potential target gene for miR‐195 (Figure 2C), and dual‐luciferase reporter gene assay (Figure 2D) showed that cotransfection with pHOXA10‐Wt led to a significantly decreased luciferase activity in the miR‐195 mimic group compared with the NC group (*P* < .05), while no significant difference was evident in the luciferase activity upon cotransfection with pHOXA10‐Mut (*P* > .05). The microarray analysis showed that LINC00461 was localized in the cytoplasm, and FISH test further identified LINC00461’s location in NCI‐H1650 cells under a fluorescence microscope (Figure 2E). The results from RIP assay and RNA pull‐down showed that LINC00461 was significantly enriched within Ago2, and LINC00461 directly bound to miR‐195 (*P* < .05) (Figure 2F‐G). The aforementioned findings demonstrated that LINC00461 up‐regulated HOXA10 by regulating miR‐195.

### 3.3 Silencing LINC00461 inhibited the expression of invasion/apoptosis‐related factors

The cells transfected with the shRNA‐NC, LINC00461 + mimic NC, sh‐LINC00461 and LINC00461 + miR‐195 mimic exhibited high expression of fluorescence, with transfection efficiency peaking to about 80% (*P* > .05). Moreover, the cells in the NC, miR‐195 mimic, miR‐195 inhibitor, sh‐HOXA10 and sh‐HOXA10 + miR‐195 inhibitor groups also revealed a high expression of fluorescence, with transfection efficiency reached about 80% (*P* < .05) (Figure S1).

**Figure 3:**
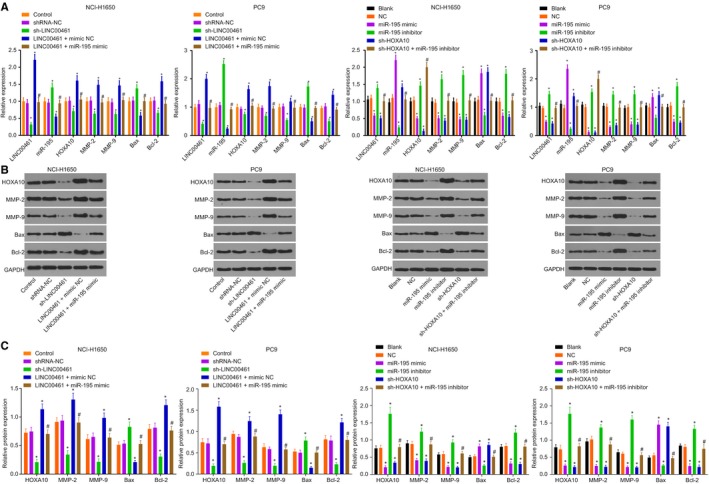
Silencing LINC00461 inhibits the expression of invasion/apoptosis‐related factors. A, The determination of RT‐qPCR demonstrates that silencing LINC00461 decreases mRNA levels of HOXA10, MMP‐2, MMP‐9 and Bcl‐2 but increases miR‐195 expression and Bax mRNA levels. B and C, The determination of Western blot analysis reveals that silencing LINC00461 decreases the protein levels of HOXA10, MMP‐2, MMP‐9 and Bcl‐2 but increases Bax protein levels. *P < .05, vs the normal group. ^#^ *P* < .05, vs the control group; D, The determination of RT‐qPCR demonstrates that the forced expression of miR‐195 decreases mRNA levels of LINC00461, HOXA10, MMP‐2, MMP‐9 and Bcl‐2 but increases Bax mRNA level. E and F, The determination of Western blot analysis demonstrates that the forced expression of miR‐195 decreases protein levels of LINC00461, HOXA10, MMP‐2, MMP‐9 and Bcl‐2 but increases Bax protein level. *P < .05, vs the blank and NC groups. ^#^ *P* < .05, vs the miR‐195 inhibitor group. The experimental data of RT‐qPCR and Western blot analysis were measurement data, expressed as mean value ± standard deviation and analysed by one‐way ANOVA. The experiment was run in triplicate independently. miR‐195, microRNA‐195; HOXA10, Homeobox A10; ANOVA, analysis of variance; RT‐qPCR, reverse transcription quantitative polymerase chain reaction; NC, negative control; MMP, Matrix metalloproteinase; Bcl‐2, Bcelllymphoma/lewkmia‐2; and Bax, Bcl‐2‐associated X protein

We initially investigated the impact on the expression of invasion/apoptosis‐related factors in NCI‐H1650 and PC9 cell lines. Among the LINC00461‐related groups (Figure 3A‐C), there was no significant difference among the control, shRNA‐NC, LINC00461 + mimic NC, sh‐LINC00461 and LINC00461 + miR‐195 mimic groups (*P* > .05). In comparison with the control group, the expression of LINC00461, HOXA10, MMP‐2, MMP‐9 and Bcl‐2 in the sh‐LINC00461 group was decreased while the expressions of miR‐195 and Bax were elevated (*P* < .05), while an opposite trend was evident in the LINC00461 + mimic NC group (*P* < .05). The results in the LINC00461 + miR‐195 mimic group indicated that miR‐195 mimic reversed LINC00461 overexpression. Among miR‐195–related groups (Figure 3D‐F), no significant difference was observed in the expression of HOXA10, MMP‐2, MMP‐9, Bcl‐2 and Bax in the blank, NC and sh‐HOXA10 + miR‐195 inhibitor groups (*P* > .05). In comparison with the blank group, the expression of miR‐195 in the miR‐195 mimic group was markedly increased, paralleled by the dramatic reduction of LINC00461 expression (*P* < .05). The Bax mRNA and protein expression in the miR‐195 mimic and sh‐HOXA10 groups were increased while the same for HOXA10, MMP‐2, MMP‐9 and Bcl‐2 were decreased in the miR‐195 mimic and sh‐HOXA10 groups (*P* < .05). No significant difference was observed in the sh‐HOXA10 + miR‐195 inhibitor group as compared to the blank group (*P* > .05). The aforementioned results were consistent in both NCI‐H1650 and PC9 cell lines. These results revealed that LINC00461 silencing could up‐regulate miR‐195 to inhibit the expression of HOXA10, thereby certainly suppressing the expression of cancer cell invasion/apoptosis‐related factors.

### 3.4 Silencing LINC00461 and overexpressed miR‐195 inhibited proliferation, migration and invasion but enhanced radiosensitivity of lung adenocarcinoma cells

**Figure 4:**
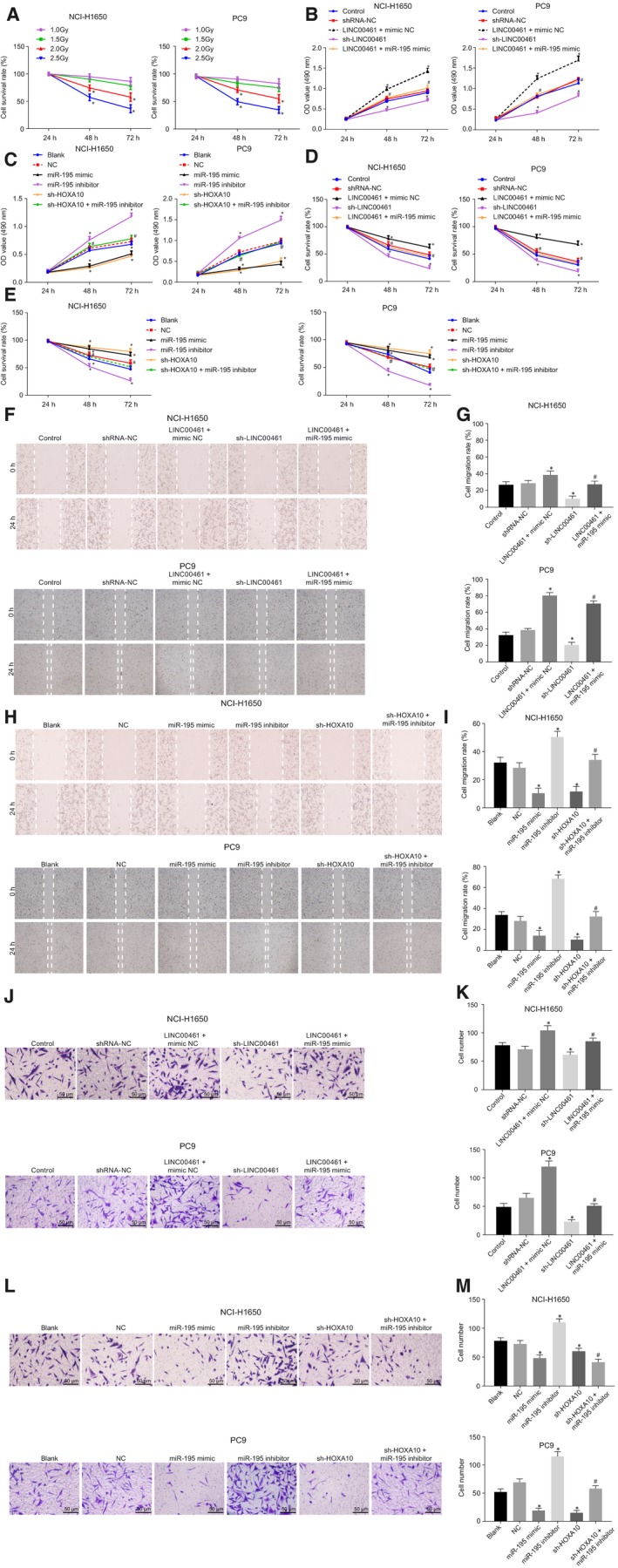
Silenced LINC00461 suppresses cell proliferation, migration and invasion abilities and enhances the radiosensitivity of lung adenocarcinoma cells via overexpression of miR‐495. A, Cell survival rate of the NCI‐H1650 and PC9 cell lines under different X‐ray doses. B, MTT assay results of cell proliferation of the NCI‐H1650 and PC9 cell lines after LINC00461 alteration. C, MTT assay results on cell proliferation of the NCI‐H1650 and PC9 cell lines after miR‐195 alteration. D, The radiosensitivity of the NCI‐H1650 and PC9 cell lines after LINC00461 alteration. E, The radiosensitivity of the NCI‐H1650 and PC9 cell lines after miR‐195 alteration. F, Scratch test results of cell migration of the NCI‐H1650 and PC9 cell lines after LINC00461 alteration (×40). G, Statistical analysis of F. H, Scratch test results of cell migration of the NCI‐H1650 and PC9 cell lines after miR‐195 alteration (×40). I, Statistical analysis of H. J, Transwell assay of cell invasion of NCI‐H1650 and PC9 cell lines after LINC00461 alteration (×200). K, Statistical analysis of J. L, Transwell assay of cell invasion of NCI‐H1650 and PC9 cell lines after miR‐195 alteration (×200). M, Statistical analysis of L; ^#^ *P* < .05, vs the blank group. The experimental data were measurement data and presented as mean value ± standard deviation. The difference in radiotherapy and MTT assay at different time points was analysed by repeated ANOVA. The difference in scratch test and transwell test was analysed by one‐way ANOVA. The experiment was run in triplicate independently. miR‐195, microRNA‐195; MTT, 3‐(4, 5‐dimethylthiazol‐2‐yl)‐2, 5‐diphenyltetrazolium bromide; NC, negative control; ANOVA, analysis of variance

The results of radiotherapy pre‐test showed (Figure 4A) that the cell survival rate of the 1.0 Gy and 1.5 Gy groups was high, indicating that the radiosensitivity was not obvious, while the survival rate of 2.5 Gy group was low, thereby suggesting that the high dose was not suitable for radiotherapy. Therefore, the 2.0 Gy group was used in the following experiments. We then continued the experiment to identify the influence on cell functions in the NCI‐H1650 and PC9 cell lines. As shown in Figure 4B‐G, among LINC00461‐related groups, there was no significant difference among the control and shRNA‐NC groups (*P* > .05). In comparison with the control group, cell proliferation, migration and invasion abilities of the sh‐LINC00461 group were decreased while the radiosensitivity was elevated (*P* < .05). A contradicting trend was observed in the LINC00461 + mimic NC group (*P* < .05). In comparison with the LINC00461 + mimic NC group, cell proliferation, migration and invasion abilities were decreased in the LINC00461 + miR‐195 mimic group while the radiosensitivity was markedly increased (*P* < .05). As shown in Figure 4H‐M, among the miR‐195–related groups, no significant difference was evident in cell proliferation, migration and invasion abilities in comparison with the blank, NC and sh‐HOXA10 + miR‐195 inhibitor groups (*P* > .05). Compared with the blank group, the radiosensitivity was increased while cell proliferation, migration and invasion abilities were decreased in the miR‐195 mimic and sh‐HOXA10 groups (*P* < .05); the miR‐195 inhibitor group demonstrated an opposite trend (*P* < .05), and co‐treatment with sh‐HOXA10 and miR‐195 inhibitor reversed the effect of miR‐195 inhibitor alone treatment (*P* < .05). The aforementioned results were consistent in both NCI‐H1650 and PC9 cell lines. Therefore, silencing of LINC00461 could suppress cell proliferation, migration and invasion abilities and enhance the radiosensitivity of lung adenocarcinoma cells via miR‐495 overexpression.

### 3.5 Silencing LINC00461 inhibited tumour formation of lung adenocarcinoma cells in nude mice

**Figure 5:**
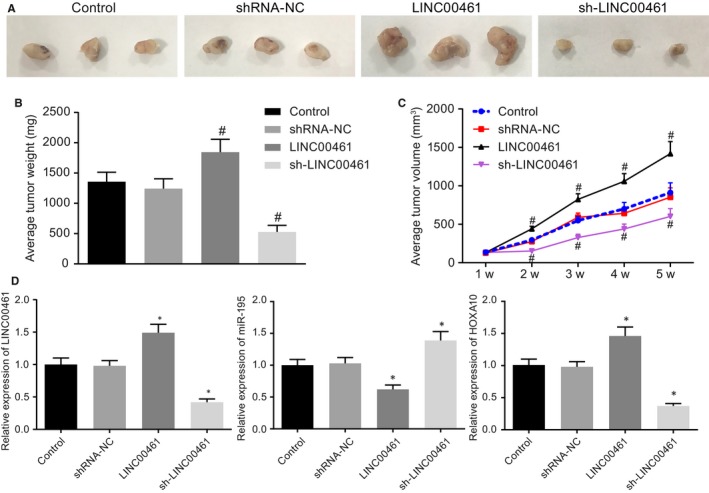
Silencing LINC00461 inhibits tumour formation of lung adenocarcinoma cells in nude mice. A, HE staining results of the tumours from nude mice after LINC00461 alteration. B, Average tumour weight of nude mice after LINC00461 alteration. C, Average tumour volume of nude mice after LINC00461 alteration. D, The expression of LINC00461, miR‐195 and HOXA10 in nude mice after LINC00461 alteration determined by RT‐qPCR. *P < .05, vs the control group. The experiment data were measurement data and presented as mean value ± standard deviation. The data were analysed by one‐way ANOVA. n = 6. The experiment was run in triplicate independently. miR‐195, microRNA‐195

HE staining results (Figure 5A‐C) showed that there were more tumour formation as time increased and there was no significant difference in the tumour volume and weight as well as in the control and the shRNA‐NC groups (*P* > .05). In comparison with the shRNA‐NC group, tumour volume and weight were decreased in the sh‐LINC00461 group while increased upon treatment with LINC00461 overexpression (Figure 5B‐E). Meanwhile, the expression of LINC00461, miR‐195 and HOXA10 was assessed in the respective tumours. We found that their expression in the shRNA‐NC and LINC00461 groups exhibited no significant changes (*P* > .05). The expression of LINC00461 and HOXA10 significantly decreased and miR‐195 expression markedly increased in the sh‐LINC00461 group when compared with the shRNA‐NC group (*P* < .05). Moreover, the expression of LINC00461 and HOXA10 was increased in the LINC00461 group while miR‐195 expression was reduced compared with the control group (Figure 5D). Thereby, the aforementioned results indicated that silencing of LINC00461 suppressed tumour formation of lung adenocarcinoma cells in nude mice.

## 4 DISCUSSION

Lung adenocarcinoma denotes the most prevalent subtypes of NSCLC affecting millions of population around the globe.^19^ Recently, the functionality of lncRNAs has been extensively reported in lung adenocarcinoma. For instance, lncRNA FEZF1‐AS1 is related to the prognosis of patients with lung adenocarcinoma as it can promote cell activities such as proliferation, migration and invasion.^20^ Therefore, the aim of our study was to explore the functional mechanism of LINC00461 in lung adenocarcinoma in relation to HOXA10. Subsequently, the key findings obtained from this study provided evidence that LINC00461 silencing could potentially suppress lung adenocarcinoma cell migration, invasion while enhancing radiosensitivity via downregulation of HOXA10 by binding to miR‐195.

Initially, our findings demonstrated that LINC00461 was up‐regulated in lung adenocarcinoma and the ectopic expression of LINC00461 stimulated lung adenocarcinoma cell proliferation, migration and invasion but inhibited radiosensitivity. LncRNA can potentially affect gene expression by regulating chromatin modifications, transcription and post‐transcriptional mechanisms and can therefore regulate cell apoptosis, proliferation, metastasis and chemo‐resistance.^21^ LncRNAs have been reported to be involved in the pathology of lung cancer.^22^ For instance, knockdown of lncRNA ENST457720 inhibits proliferation of NSCLC cells via in vivo and in vitro mechanisms.^23^ LINC00707 overexpression promotes cell proliferation and migration in lung adenocarcinoma by regulating Cdc42.^24^ LINC00461, a member of lncRNAs, is located at an intergenic region of the human chromosome 5 between two protein‐coding genes MEF2C and TMEM161B, respectively.^7^ LINC00461 has been reported to be one of the significantly up‐regulated lncRNAs in glioblastomas and metastatic prostate cancers.^25^ Yang *et al* further demonstrated that LINC00461 was critical for glioma development because of its role in affecting cell proliferation, migration and invasion through involvement of the MAPK/ERK and PI3K/AKT signalling pathways,^7^ which was in consistency with our results.

In the following experiments, miR‐195 was observed to be down‐regulated but HOXA10 was up‐regulated in lung adenocarcinoma. In addition, lung adenocarcinoma cells showed decreased HOXA10 and increased miR‐195 following LINC00461 silencing. When miR‐195 expression was decreased by inhibitor, HOXA10 expression as well as cell proliferation, invasion, migration and radiosensitivity were promoted. MiR‐195 expression has been reported to be often decreased in various types of cancers, such as hepatocellular carcinoma.^10^ In addition, a prior study highlighted the functionality of miR‐195 as a tumour suppressive miRNA in NSCLC.^26^ Besides, our findings also exhibited that LINC00461 directly regulated miR‐195. Yu et al demonstrated that overexpressed miR‐195 inhibited the growth of NSCLC cells by regulating cell cycle progression, apoptosis and senescence partially through targeting CCND3 and BIRC5 and that dysregulation of the miR‐195/BIRC5 axis contributed to the progression of lung cancer.^27^ Gao et al also reported that HMGA2, regulated by miR‐195, is involved in the proliferation, metastases and epithelial‐to‐mesenchymal transition in lung cancer.^28^ Furthermore, Liu et al reported a lower miR‐195 expression profile in NSCLC tissues and also emphasized its relevance with poor survival outcome.^11^ In addition, we found that HOXA10 was a putative target gene of miR‐195 according to the evidence in the online analysis software RNA22. Almeida et al indicated that miR‐195 inhibited HOXA10 mRNA level.^29^ Ye et al also found HOXA10 to be a potential candidate target gene of miR‐195 using Targetscan.^30^ HOXA10 has been identified to be increased in human lung cancer cells and tissues with respect to normal lungs.^14^ HOXA10 is also considered as the positive risk parameter of lung adenocarcinoma survival.^13^

In the current study, our gathered evidence supported that down‐regulated LINC00461 resulted in reduced expression of Bcl‐2. Deng M and his group reported similar results in a study based on multiple myeloma.^31^ We also gathered evidence implicating that the down‐regulated LINC00461 enhanced radiosensitivity, which is consistent with the findings of a previous study demonstrating that the lower levels of Bcl‐2 and higher levels of Bax were indicative of enhanced radiosensitivity.^32^ In addition, inhibition of MMP‐2 was also found to enhance radiosensitivity in lung cancer cells.^33^

## 5 CONCLUSION

Our study showed that LINC00461 functioned as a competing endogenous RNA to regulate HOXA10 expression by binding to miR‐195 in lung adenocarcinoma cells. Furthermore, silencing of LINC00461 and restoration of miR‐195 expression inhibited cell growth and induced apoptosis in lung adenocarcinoma cells. Altogether, our findings provided an insight on the potential of LINC00461 as a sensitizer for lung adenocarcinoma radiotherapy. Nevertheless, these data should be further validated in independent cohorts and prospective trials. In addition, further studies are still required to study whether miR‐195 and LINC00461 expressions are associated with the presence of KRAS and EGFR in lung adenocarcinoma and to better elucidate the modulatory role of KRAS and EGFR in NSCLC.

## CONFLICT OF INTEREST

The authors declare that they have no conflicts of interest concerning this study.

## AUTHORS CONTRIBUTION

JQH participated in the study design and experimental work. YJW, HMZ and XDL participated in sample collection and data analysis. XQX and YXH performed the statistical analysis and preparation of figures and table. YJW and XDL drafted the paper. All authors approved the final manuscript.

## Data Availability

The data used to support the findings of this study are available from the corresponding author upon request.
