# Corrigendum

**DOI:** 10.1111/jcmm.15416

**Published:** 2020-07-09

In Hou et al,^1^ the published article contains errors in Figure 4L. The correct figures are shown below. The authors confirm all results and conclusions of this article remain unchanged.

**Figure 4:**
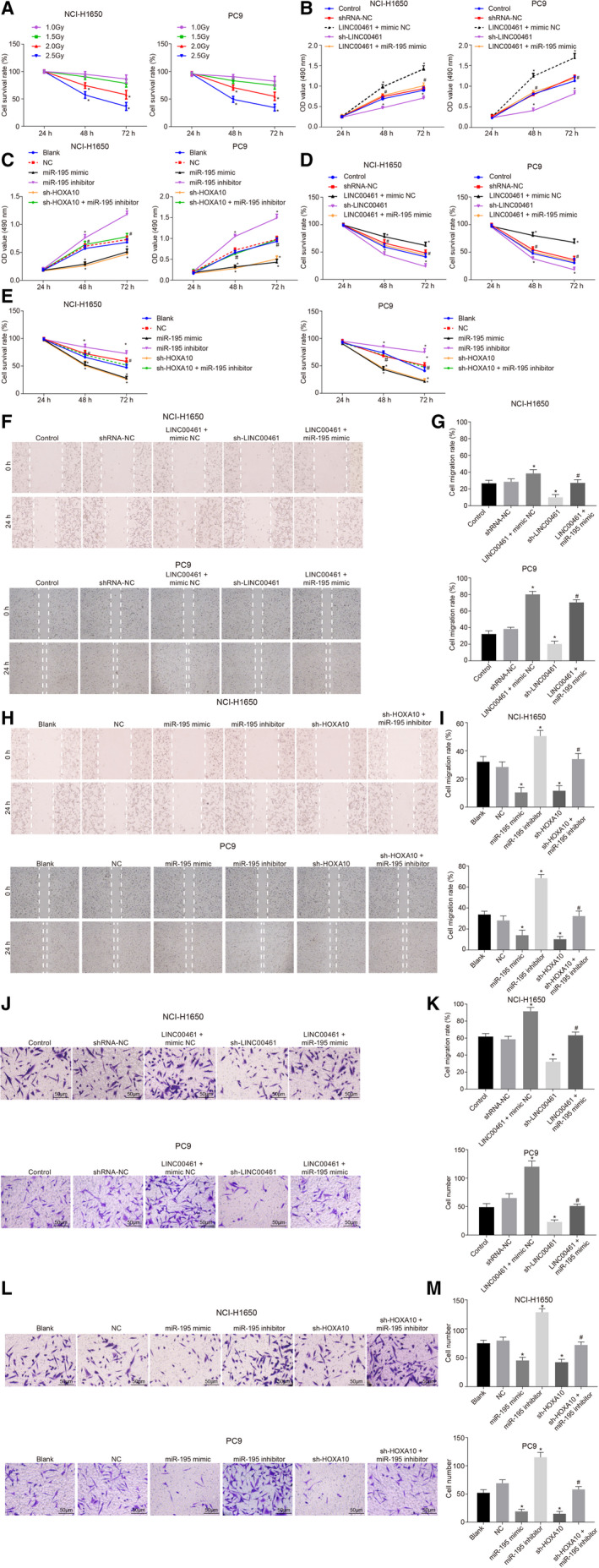
Silenced LINC00461 suppresses cell proliferation, migration and invasion abilities and enhances the radiosensitivity of lung adenocarcinoma cells via overexpression of miR‐495. A, Cell survival rate of the NCI‐H1650 and PC9 cell lines under different X‐ray doses. B, MTT assay results of cell proliferation of the NCI‐H1650 and PC9 cell lines after LINC00461 alteration. C, MTT assay results on cell proliferation of the NCI‐H1650 and PC9 cell lines after miR‐195 alteration. D, The radiosensitivity of the NCI‐H1650 and PC9 cell lines after LINC00461 alteration. E, The radiosensitivity of the NCI‐H1650 and PC9 cell lines after miR‐195 alteration. F, Scratch test results of cell migration of the NCI‐H1650 and PC9 cell lines after LINC00461 alteration (×40). G, Statistical analysis of F. H, Scratch test results of cell migration of the NCI‐H1650 and PC9 cell lines after miR‐195 alteration (×40). I, Statistical analysis of H. J, Transwell assay of cell invasion of NCI‐H1650 and PC9 cell lines after LINC00461 alteration (×200). K, Statistical analysis of J. L, Transwell assay of cell invasion of NCI‐H1650 and PC9 cell lines after miR‐195 alteration (×200). M, Statistical analysis of L; #*P* < .05, vs the blank group. The experimental data were measurement data and presented as mean value ± standard deviation. The difference in radiotherapy and MTT assay at different time points was analysed by repeated ANOVA. The difference in scratch test and transwell test was analysed by one‐way ANOVA. The experiment was run in triplicate independently. miR‐195, microRNA‐195; MTT, 3‐(4, 5‐dimethylthiazol‐2‐yl)‐2, 5‐diphenyltetrazolium bromide; NC, negative control; ANOVA, analysis of variance
